# Genetic landscape of early-onset dementia in Hungary

**DOI:** 10.1007/s10072-022-06168-8

**Published:** 2022-06-25

**Authors:** Dora Csaban, Anett Illes, Toth-Bencsik Renata, Peter Balicza, Klara Pentelenyi, Viktor Molnar, Andras Gezsi, Zoltan Grosz, Aniko Gal, Tibor Kovacs, Peter Klivenyi, Maria Judit Molnar

**Affiliations:** 1 Institute of Genomic Medicine and Rare Disorders, Semmelweis University, 1082 Budapest, Hungary; 2 PentaCore Laboratory Budapest, Budapest, Hungary; 3 Department of Measurement and Information Systems, Budapest University of Technology and Economics, Budapest, Hungary; 4 Department of Neurology, Semmelweis University, Budapest, Hungary; 5 Department of Neurology, Faculty of Medicine, Albert Szent-Györgyi Clinical Center, University of Szeged, Szeged, Hungary

**Keywords:** Early-onset dementia, Alzheimer’s disease, Frontotemporal dementia, Next-generation sequencing, Genetic risk, Monogenic forms

## Abstract

**Introduction:**

Early-onset dementias (EOD) are predominantly genetically determined, but the underlying disease-causing alterations are often unknown. The most frequent forms of EODs are early-onset Alzheimer’s disease (EOAD) and frontotemporal dementia (FTD).

**Patients:**

This study included 120 Hungarian patients with EOD (48 familial and 72 sporadic) which had a diagnosis of EOAD (*n* = 49), FTD (*n* = 49), or atypical dementia (*n* = 22).

**Results:**

Monogenic dementia was detected in 15.8% of the patients. A pathogenic hexanucleotide repeat expansion in the *C9ORF72* gene was present in 6.7% of cases and disease-causing variants were detected in other known AD or FTD genes in 6.7% of cases (*APP*, *PSEN1*, *PSEN2*, *GRN*). A compound heterozygous alteration of the *TREM2* gene was identified in one patient and heterozygous damaging variants in the *CSF1R* and *PRNP* genes were detected in two other cases. In two patients, the coexistence of several heterozygous damaging rare variants associated with neurodegeneration was detected (1.7%). The *APOE* genotype had a high odds ratio for both the *APOE* ɛ4/3 and the ɛ4/4 genotype (OR = 2.7 (95%CI = 1.3–5.9) and OR = 6.5 (95%CI = 1.4–29.2), respectively). In *TREM2*, *SORL1*, and *ABCA7* genes, 5 different rare damaging variants were detected as genetic risk factors. These alterations were not present in the control group.

**Conclusion:**

Based on our observations, a comprehensive, targeted panel of next-generation sequencing (NGS) testing investigating several neurodegeneration-associated genes may accelerate the path to achieve the proper genetic diagnosis since phenotypes are present on a spectrum. This can also reveal hidden correlations and overlaps in neurodegenerative diseases that would remain concealed in separated genetic testing.

**Supplementary Information:**

The online version contains supplementary material available at 10.1007/s10072-022-06168-8.

## Introduction

In early-onset dementia (EOD), the cognitive and functional decline starts before the age of 65. In the background of EOD, early-onset Alzheimer’s disease (EOAD) and frontotemporal dementia (FTD) are the most frequent types [1]. EOAD is characterized biologically by two hallmark proteinopathies: extraneuronal amyloid plaques and intraneuronal neurofibrillary tangles composed of beta-amyloid peptide and hyperphosphorylated tau protein, respectively [2]. Abnormal protein deposition occurs over decades and leads to neurodegeneration and significant subsequent cognitive decline, ultimately leading to death. The heritability of EOAD is significantly higher than in late-onset Alzheimer’s disease (LOAD) [2]. However, the investigation of the most frequently known monogenic autosomal dominantly inherited genes, associated with EOAD (*APP*, *PSEN1*, *PSEN2*) in 90–95% of the cases, could not clearly explain the genetic background [2]. Previous genome-wide association studies (GWAS), whole-exome (WES), and whole-genome sequencing (WGS) studies have revealed genetic susceptibility factors to Alzheimer’s disease (AD). The most significant genetic risk factor is *APOE* ɛ*4. APOE* ɛ*4* has an onset-modifying effect in EOAD patients carrying a pathogenic mutation in other AD-associated genes [2]. Further relevant risk factors are some rare variants in *SORL1*, *TREM2*, and *ABCA7* [1]

FTD, the second most common type of EOD after AD, represents a wide pathological spectrum with different proteinopathies. In FTD patients, progressive deterioration of personality, behavioral changes, language changes, and cognitive deficits manifest due to degeneration of the frontal and temporal lobes. A significant proportion of patients with FTD spectrum disorders display either dementia or other signs of neurodegenerative disorders in their families (~ 40%). Moreover, 10–30% of FTD cases show autosomal dominant inheritance which also highlights the importance of genetic investigation in FTD patients. Currently, more than 50 genes are associated with FTD, amyotrophic lateral sclerosis-FTD (ALS-FTD), and FTD-parkinsonism phenotypes. The most common disease-causing alteration is the hexanucleotide repeated expansion (HRE) of the *C9ORF72* gene, while the most frequently studied genes are *MAPT*, *GRN*, and *FUS*. In recent years, variants of the *TARDBP*, *SQSTM1*, *TREM2*, *CSF1R*, and *TMEM106B* genes have also been described as monogenic and/or disease-modifying factors in FTD [1, 3, 4].

Dementia as a symptom was described in association with several other neurodegenerative disorders such as Huntington’s disease, Parkinson’s disease (PD), ALS, and Creutzfeldt-Jakob disease (CJD). Clinical and pathological studies identified substantial similarities among these disorders. Some authors suggested that this may be also present on the genetic level. Due to clinical and neuropathological overlaps with other dementias, AD is misdiagnosed in 17–30% of patients [5]. Therefore, the examination of different types of dementia-associated genes in patients with atypical symptoms and with early onset is essential for identifying disease-causing alterations and understanding the pathomechanism. Comprehensive genetic studies determining the genetic profile of neurodegenerative disorders might contribute to the establishment of the optimal diagnostic workflow in order to support the medical decision for an accurate diagnosis and further management of the patient.

## Patients and methods

### Patients

Altogether, 120 Hungarian patients with EOD (the mean age of onset (AOO): 56.05 ± 6.95 years) were included in our study based on clinical data (Table 1). Each patient was diagnosed by a board-certified neurologist. EOAD was diagnosed in 49 cases, early-onset FTD in 49 cases, and atypical dementia in 22 cases. In the latter group (atypical dementias), ten patients had a positive family history. In our cohort, the case was defined as familial if the neurodegenerative disorder was diagnosed among first or second-degree relatives. In 42 out of the 48 familial cases, first-degree relatives suffered from symptoms of dementia or other neurodegenerative disorders. Patients having classical vascular dementia or typical familial CJD have been excluded. The investigated patients were selected from the disease-specific biobank (NEPSYBANK) of our institute between 2009 and 2020 [6]. Controls having WES or WGS data were selected from our NEPSYBANK and NEGTAR (biobank of aged healthy persons) [6, 7]. From our biobanks, 55 healthy subjects and 82 patients without any neurodegenerative symptoms were selected as controls. The mean age of all controls was 52.93 ± 17.88 years. NEPSYBANK included only a few healthy individuals, so patients with neuromuscular disease were also included in our control group. The inclusion criteria included the absence of signs/symptoms of normal cognition and neurodegeneration. Before sampling and molecular genetic analysis, both patients and control subjects signed a written informed consent form in accordance with the Declaration of Helsinki. The study was confirmed by the Hungarian Scientific and Research Ethical Committee (No.44599–2/2013/EKU). In all assessed cases, molecular genetic analysis was performed for diagnostic purposes.

**Table 1:** Patients with early-onset dementia

	Alzheimer’s disease		FTD		Atypical dementias	
	Number of familial cases (mean AOO ± SD)	Number of sporadic cases (mean AOO ± SD)	Number of familial cases (mean AOO ± SD)	Number of sporadic cases (mean AOO ± SD)	Number of familial cases (mean AOO ± SD)	Number of sporadic cases (mean AOO ± SD)
Female	15 (55.7 ± 7.7)	18 (54.2 ± 14.2)	12 (54.9 ± 5.6)	13 (57.1 ± 6.1)	7 (57.9 ± 5.43)	4 (53.3 ± 9.4)
Male	6 (58.2 ± 26.4)	10 (52.6 ± 18.7)	5 (57.4 ± 2.4)	19 (54.49 ± 6.03)	3 (58.0 ± 1.73)	8 (58.9 ± 4.8)

*AOO*, age of onset; *SD*, standard deviation; *FTD*, frontotemporal dementia

### Molecular genetic analysis

In each diagnostic method, DNA was extracted from blood using the QIAamp DNA blood kit, according to the manufacturer’s instructions (QIAgen, Hilden, Germany). The most common AD genetic risk factor, *APOE* genotype, was screened for by the RFLP (restriction fragment length polymorphism) method. The *C9ORF72* HRE was investigated by Amplidex® PCR/CE C9orf72 kit (Asuragen, Inc., Austin). Repeat numbers exceeding 30 were considered positive based on the literature. In all cases, this was the first molecular genetic test. As a second step, the coding regions of *PSEN1*, *PSEN2*, *APP*, *MAPT*, and *GRN* genes were investigated with Sanger sequencing using a 3500 Series Genetic Analyzer (Applied Biosystems, Foster City, USA). In autosomal dominant EODs, the *PRNP* gene was investigated as well if the previous genetic tests were normal. The sequences were compared with the human reference genome (GRCh37/hg19) by using the NCBI’s Blast® application.

In 38 cases, targeted next-generation sequencing (NGS) panel sequencing was performed. Altogether, 127 genes were selected for investigation (Supplementary Table). These genes were previously associated with monogenic dementias or other neurodegenerative disorders such as PD, ALS, and NBIA (neurodegeneration with brain iron accumulation). The targeted NGS panel was investigated by SureSelect^QXT^ Target Enrichment for the Illumina Platform (Agilent Technologies, Santa Clara, CA, USA) according to the protocol. Library preparation was followed by NGS using MiSeq Reagent Kit v3 (600 cycles) for sequencing on MiSeq (Illumina, San Diego, CA, USA). WES was performed in 16 probands. A DNA library preparation was performed using Agilent SureSelect^QXT^ Human All Exon v5 reagents according to the manufacturer’s instructions. Library preparation was followed by NGS by using Illumina HiSeq PE Cluster Kit v4 for cluster generation on cBot, HiSeq SBS Kit v4 for sequencing on the HiSeq2500 system (Illumina, San Diego, CA, USA) [8]. Segregation analysis was performed in cases where the proband’s family members agreed.

### Bioinformatics analysis

Prior to the variant calling, the raw NGS data was qualitatively filtered and sequences were aligned to the GRCh37/hg19 reference genome based on the default parameters of BWA-MEM (Burrows-Wheeler Aligner; version 0.7.15) [9]. Annotated variants of the variant call format (VCF) files were filtered using VariantAnalyzer software developed by the Budapest University of Technology and Economics. Filtration for potentially rare damaging variants (RDVs) was prepared based on the SnpEff software [10], ClinVar database [11], and several population databases such as the Genome Aggregation Database (gnomAD v2.1). The classification of the rare non-synonymous variants was determined by following the American College of Medical Genetics and Genomics (ACMG) guidelines [12]. Interpretation of novel rare alterations was performed by using Franklin applications [13]. The frequency of the interpreted pathogenic variants, likely pathogenic alterations and variants with uncertain significance (VUS), was examined in our control group.

## Results

In our study, 120 unrelated patients with EOD were analyzed from which positive family history for neurodegenerative disorders was detected in 48 cases. Abnormal *C9ORF72* HRE was identified in 8 cases (Table 2). In the investigated genes, we identified 22 probable RDVs. These alterations were classified as 8 pathogenic, 6 likely pathogenic, or 8 VUS according to ACMG (Tables 3, 4, and 5).

### The identified monogenic dementias

#### C9ORF72 hexanucleotide repeat expansion

Since the presence of *C9ORF72* HRE is common in FTD patients, we screened this alteration in all patients before starting the sequencing studies. In 8/120 cases, pathogenic *C9ORF72* HRE was found. In the positive cases, the mean AOO was 54.63 ± 6.9 years, and the family history was positive in six cases. Seven patients had either parkinsonism (P45), hallucinations (P3, P24, P45), or symptoms of motor neuron disease, beside the typical FTD features (Table 2).

**Table 2:** Patients with *C9orf72* hexanucleotide repeat expansion

Patient ID	Form	AOO	Sex	Symptoms	Clinical diagnosis
P3	F	56	f	Memory impairment, apathia, depression, executive disabilities, hallucination, urinary incontinence	FTD
P15	F	56	f	Memory deficit, aphasia, paraphasia	FTD
P17	S	50	m	Memory impairment, aphasia, dyscalculia, delusion	FTD
P24	S	40	f	Memory deficit, anxiety, depression, hallucination	FTD
P32	F	55	f	Memory deficit, anxiety, depression,	AD
P45	F	62	f	Severe cognitive impairment, parkinsonism, hallucination	FTD
P80	F	60	m	muscle weakness, dysphagia, spasticity, pyramidal signs, cognitive impairment, aphasia	FTD/MND
P107	F	58	f	Cognitive impairment, apathia	FTD

*F*, familial; *S*, sporadic; *AOO*, age of onset; *f*, female; *m*, male; *FTD*, frontotemporal dementia; *AD*, Alzheimer’s disease; *MND*, motor neuron disease

#### Rare damaging variants in AD- or FTD-associated genes

In this group, RDVs were identified in 8 patients. Four of them had a positive family history. The clinical characteristics of our patients are presented in Table 3. The mean AOO in patients with RDVs was 50.25 ± 8.4 years. From these variants, 3 were described as pathogenic in ClinVar. The other RDVs were pathogenic or likely pathogenic according to ACMG. The *APP* p.V717F pathogenic substitution resulted in deterioration of short-term memory, serious language impairment, epileptic seizure, myoclonus-like jerks, hypokinesis, and bradykinesis starting at age 40 [14]. Two pathogenic variants were identified in the *GRN* gene (P11: c.708 + 1G > A; P117: p.Ser226TrpfsTer28), and the symptoms of P11 and P117 were similar to those that were previously described with these alterations [15, 16]. In two unrelated patients, a splice variant in the *GRN* gene (c.264 + 2 T > C) was detected which was classified as likely pathogenic based on the ACMG. This variant has been previously described in association with FTD [16]. In both cases, symptoms developed at a similar age and the clinical phenotype was largely overlapping (i.e., aphasia, amotivation, severe cognitive impairment). In both patients, mild white matter lesions had been detected in addition to the predominant frontal lobe atrophy. In the *PSEN1* gene, three RDVs were detected in our cohort. Interestingly, in a female patient carrying the p.G206S variant, the symptoms started early in her 30s when her brain MRI was still normal, while bilateral symmetric hippocampal atrophy developed 2 years later. In P111 and his father carrying the *PSEN1* p.L166R variant, the disease started with paraspasticity. The symptoms and course of the disease in the patient carrying the *PSEN1* p.V89L variant were similar to those already described in the literature [17]. Ochalek et al. reported our patient’s iPSC line with elevated TAU phosphorylation, increased amyloid-β 1–40 (Aβ_1–40_) and amyloid-β 1–42 (Aβ_1–42_) levels and a significantly different Aβ_1–42_/Aβ_1–40_ ratio from control cell lines [18]. An AD-associated variant was detected in only one case in the *PSEN2* gene (0.83%). The p.S130L variant was suggested to be a risk factor [19].

**Table 3:** Disease-causing variants of identified monogenic dementias (except *C9ORF72*)

Patient ID	Form	AOO	Sex	Symptoms	Gene	Variant ID	Zygosity	Clinical significance	ACMG classification	MAF (non-neuro)	Patients	Controls	Reference
P59	F	40	f	Severe short-term memory impairment; serious language impairment, epileptic seizure, myoclonus-like jerks, hypokinesis	*APP*	p.V717F c.2149G > T rs63750264	het	P	P	-	1/120	0/137	[14]
P11	S	60	f	Behavioral changes; speech deterioration; severe aphasia; rigidity; sever dysphagia	*GRN*	c.708 + 1G > A rs63749817	het	P/LP	P	-	1/120	0/137	[15]
P117	S	49	m	Behavioral changes, aphasia	*GRN*	p.Ser226TrpfsTer28 c.675_676delCA rs63751085	het	P	P	-	1/120	0/137	[16]
P10	S	58	f	Aphasia, apathy, severe cognitive impairment, echolalia	*GRN*	c.264 + 2 T > C	het	D	LP	-	2/120	0/137	[15]
P46	F	59	m	Aphasia, apathy, severe cognitive impairment									
P27	F	52	f	Short-term memory impairment, anxiety, amnestic, and executive disabilities	*PSEN1*	p.V89L c.265G > C	het	D	LP	-	1/120	0/137	[17]
P31	S	39	f	Memory impairment, disorientation, hallucination, psychotic sessions, conversion, mixed dissociative disorder, myoclonus, impaired speech, and apraxia	*PSEN1*	p.G206S c.616G > A rs63750569	het	D	LP	-	1/120	0/137	[44]
P111	F	45	f	Spastic paraparesis, dysarthria, dysphagia, severe cognitive decline, and progressive loss of speech	*PSEN1*	p.L166R c.497 T > G rs63750265	het	D	LP	-	1/120	0/137	[27]
P56	S	52	m	Memory impairment, progressive dysphagia, apraxia, tetrapyramidal signs, spasticity, and urinary incontinence	*CSF1R*	c.2646_2654 + 6del	het	D	P	-	1/54	0/137	-
P112	F	29	m	learning difficulty, concentration problems, gait disturbance, speech deterioration	*PRNP*	7- OPRI	het	D	P	< 0.01	1/120	0/137	[22]
P14	S	51	m	Behavioral changes, memory deficit, mixed aphasia, apathy, epileptic seizures, rigor, resting tremor, urinary incontinence	*TREM2*	p.R47C c.139C > T rs753325601	comp. het	D	VUS	< 0.01	1/54	0/137	[21]
						p.A105Rfs*84 c.313delG rs386834141		P	P	< 0.01	1/54	0/137	[20]

From our biobanks, 55 healthy subjects and 82 patients without any neurodegenerative symptoms were selected as controls. *F*, familial; *S*, sporadic; *AOO*, age of onset; *f*, female; *m*, male; *het.*, heterozygous; *comp. het.*, compound heterozygous; *D*, damaging; *P*, pathogenic; *LP*, likely pathogenic; *VUS*, variants with uncertain significance; *MAF*, minor allele frequency

#### Monogenic dementias associated with other neurodegenerative disorders

Although *TREM2* heterozygous RDVs were reported as risk factors for AD and FTD, the biallelic rare variants were associated with a monogenic disease, Nasu-Hakola disease (NHD). In P14 the p.A105Rfs* and p.R47C of *TREM2* were identified in a compound heterozygous form. The p.A105Rfs* substitution has already been published in NHD as a likely pathogenic alteration in the homozygous form [20]. The p.R47C alteration is a VUS according to ACMG but it was previously described in a homozygous form in a patient with behavioral variant FTD without bone cysts [21]. Our patient’s symptoms started at the age of 51 with apathy, anhedonia, depressed mood, and mild short-term memory deficit. Three years after the onset of dementia, the first epileptic seizure occurred, during which severe bilateral hippocampal and cortical atrophy was seen on MRI. In addition to memory loss, dyscalculia appeared a few years later. Abstract thinking and verbal fluency were also severely affected. Five years later, mixed-type aphasia developed. We assume that this patient has a *TREM2*-associated monogenic form of the disease.

In two families with EOD, rare heterozygous alterations were found in autosomal dominant inherited genes that were primarily associated with other neurodegenerative disorders. The pathogenic c.2646_2654 + 6del variant was identified in the *CSF1R* gene (P56), which was previously associated with autosomal dominant leukoencephalopathy (adult-onset leukoencephalopathy with axonal spheroid and pigmented glia—ALSP) [3]. This novel variant was classified as a pathogenic alteration according to ACMG. P56 was referred to us with the clinical diagnosis of FTD. The patient’s symptoms started at the age of 52 with word-finding difficulty, memory impairment, progressive dysphagia, apraxia, tetrapyramidal signs, spasticity, and incontinence. His previous medical history was reviewed in light of the genetic finding and a mild bilateral white matter lesion was detected during a re-evaluation of the brain MRI.

A disease-causing variant in the *PRNP* gene, which was associated with CJD, was also detected. In P112 a 7-octapeptide repeat insertion was identified. His symptoms began at the age of 29 with a learning difficulty, concentration problems, and gait disturbance. The proband’s cognitive decline and relatively slow disease progression in addition to his symptoms resemble the previously published cases with 168 bp insertion in the *PRNP* gene [22].

### Genetic risk factors associated with AD

*APOE* genotype testing detected a high odds ratio for both the *APOE* ɛ4/3 and the ɛ4/4 genotype (OR = 2.7 (95%CI = 1.3–5.9); OR = 6.5 (95%CI = 1.4–29.2)). In *TREM2*, *SORL1*, and *ABCA7* genes, 5 different risk RDVs were detected in our cohort while these alterations were not present in the control group (Table 4). The mean age of the patients carrying these variants was 61.00 ± 4.24 years, and the phenotypic characteristics of these patients were shown in Table 4. In our Hungarian population, a heterozygous *TREM2* alteration, p.R47H was identified in 2 patients (1.6%).

In the *ABCA7* gene, two heterozygous missense RDVs (p.R1228C and p.D1957Y) and a rare damaging heterozygous nonsense mutation (p.Y750*) were found (P26, P35, and P75). These variants were absent in the control group. The *SORL1* p.K2044R RDV was identified in one case (P33) and was missing from both the control group and the gnomAD database. Despite the scarce information on the detected variants in *ABCA7* and *SORL1*, both of these could potentially contribute to the increased risk of AD [23].

**Table 4:** Previously described susceptibility genes associated with AD/FTD (except *APOE* ɛ*4*)

Patient ID	Form	AOO	Sex	Symptoms	Gene	Variant ID	Zygosity	Clinical significance	ACMG classification	MAF (non-neuro)	Patients	Controls	Reference
P45	F	62	f	Severe cognitive impairment	*TREM2*	p.R47H c.140G > A rs75932628	B	RF	VUS	0.025	2/54	0/137	[34]
P71	S	53	m	Memory impairment, disorientation, amnestic and executive disabilities									
P26	F	65	f	Behavioral changes, mixed aphasia, visuospatial disturbancy	*ABCA7*	p.R1228C c.3682C > T rs201115102	het	D	VUS	< 0.01	1/54	0/137	-
P35	S	61	m	Short-term memory impairment, visuospatial disturbancy, dyscalculia, apraxia	*ABCA7*	p.D1957Y c.5869G > T	het	D	VUS	-	1/54	0/137	-
P75	S	61	m	Vertical gaze pulsy, rigor, ataxia, severe memory impairment	*ABCA7*	p.Y750* c.2250C > A rs757657653	het	D	LP	< 0.01	1/54	0/137	[33]
P33	F	64	m	Cognitive deficit, visuospatial disorientation,	*SORL1*	p.K2044R c.6131A > G	het	D	VUS	-	1/54	0/137	-

From our biobanks, 55 healthy subjects and 82 patients without any neurodegenerative symptoms were selected as controls. *F*, familial; *S*, sporadic; *AOO*, age of onset; *f*, female; *m*, male; *het.*, heterozygous; *B*, benign; *RF*, risk factor; *D*, damaging; *P*, pathogenic; *LP*, likely pathogenic; *VUS*, variants with uncertain significance; *MAF*, minor allele frequency

### Alterations in genes associated with other neurodegenerative disorders

There are several genes in which alterations are associated with different neurodegenerative disorders, such as PD, NBIA, and ALS [5]. In most of them, cognitive decline is an important coexisting clinical sign.

In four patients, rare heterozygous RDVs were detected in other neurodegeneration-associated genes, such as *PRKN* (PD), *LRRK2* (PD), *PARK7* (PD), *C19ORF12* (NBIA), *SPG11* (Spastic paraplegia-SP/ALS), and *PSAP* (Metachromatic leukodystrophy-MLD) (Table 5). Neither of these variants were detected in our control group nor in the gnomAD database except *PRKN* p.T240M, *SPG11* p.V2053M, and *PSAP* p.E108V, which were present at a low frequency (< 0.001).

#### Heterozygous alterations in autosomal recessive inherited genes

In two cases (P35 and P50), coexisting rare heterozygous alterations were observed (Table 6). In patient P50, besides the *PARK7* p.L92Tfs* nonsense substitution, one further RDV was present in *C19ORF12* (p.P60A). The substitutions had not been described previously. Their ACMG classification was likely pathogenic and pathogenic consequently. This patient had some distinctive features such as parkinsonism, short-term memory failure, prefrontal symptoms, and visuospatial deficit, which could be explained by the interaction of these genes. In P35, three potentially heterozygous RDVs were identified (*ABCA7*-D1957Y, *LRRK2*-A1862V, and *SPG11*-V2053M [24]). In P81, a single-heterozygous RDV was observed in the *PRKN* (T240M) gene [25]. In P52, the RDV E108V was found within the *PSAP* gene. The clinical phenotypes of these patients were shown in Table 5.

**Table 5:** Heterozygous damaging rare variants associated with other autosomal recessive inheritance neurodegenerative disorders

Patient ID	Form	AOO	Sex	Symptoms	Gene	Variant ID	Zygosity	Clinical significance	ACMG classification	MAF (non-neuro)	Inheritance	Patients	Controls	Reference
P50	F	60	m	Short-term memory impairment; parkinsonism, prefrontal symptoms	*C19ORF12*	p.P60A c.178C > G	het	D	LP	-	AR, AD	1/54	0/137	-
P81	S	59	m	Behavioral changes, anxiety	*PRKN*	p.T240M c.719C > T rs137853054	het	Conf. Of P	P	< 0.01	AR	1/54	0/137	[25]
P35	S	61	m	Short-term memory impairment, visuospatial disturbancy, dyscalculia, apraxia	*LRRK2*	p.A1862V c.5585C > T	het	D	VUS	-	AD	1/54	0/137	-
P50	F	60	m	Short-term memory impairment, parkinsonism, prefrontal symptoms	*PARK7*	p.Ile91_Leu92fs c.273_274insA	het	D	P	-	AR	1/54	0/137	-
P35	S	61	m	Short-term memory impairment, visuospatial disturbancy, dyscalculia, apraxia	*SPG11*	p. V2053M c.6157G > A rs149003934	het	VUS	VUS	< 0.01	AR	1/54	0/137	[24]
P52	F	48	f	Depression, memory impairment	*PSAP*	p.E108V c.323A > T rs763295469	het	D	VUS	< 0.01	AR	1/54	0/137	-

From our biobanks, 55 healthy subjects and 82 patients without any neurodegenerative symptoms were selected as controls. *F*, familial; *S*, sporadic; *AOO*, age of onset; *f*, female; *m*, male; *het.*, heterozygous; *D*, damaging; *Conf. Of P.*, conflicting interpretations of pathogenicity; *P*, pathogenic; *LP*, likely pathogenic; *VUS*, variants with uncertain significance; *AR*, autosomal recessive; *AD*, autosomal dominant; *MAF*, minor allele frequency

**Table 6:** Coexisting rare heterozygous alterations

Patient ID	Form	AOO	Sex	Symptoms	Gene	Variant ID	Zygosity	Clinical significance	ACMG classification	MAF (non-Finnish)	Inheritance	Patients	Controls	Reference
P50	F	60	m	Short-term memory impairment, prefrontal symptoms, parkinsonism	*C19ORF12*	p.P60A c.178C > G	het	D	LP	-	AR, AD	1/54	0/137	-
					*PARK7*	p.Ile91_Leu92fs c.273_274insA	het	D	P	-	AR	1/54	0/137	-
P35	S	61	m	Short-term memory impairment, visuospatial disturbancy, dyscalculia, apraxia	*ABCA7*	p.D1957Y c.5869G > T	het	D	VUS	-	AD	1/54	0/137	-
					*LRRK2*	p.A1862V c.5585C > T	het	D	VUS	-	AD	1/54	0/137	-
					*SPG11*	p.V2053M c.6157G > A rs149003934	het	VUS	VUS	< 0.01	AR	1/54	0/137	[24]

From our biobanks, 55 healthy subjects and 82 patients without any neurodegenerative symptoms were selected as controls. *F*, familial, *S*; sporadic; *AOO*, age of onset; *f*, female; *m*, male; *het.*, heterozygous; *D*, damaging; *P*, pathogenic; *LP*, likely pathogenic; *VUS*, variants with uncertain significance; *AR*, autosomal recessive; *AD*, autosomal dominant; *MAF*, minor allele frequency

## Discussion

This study is the first comprehensive genetic testing targeting the genetic background of early-onset dementias in Hungary. A monogenic disease was identified in the background of 15.8% of the cases investigated in our cohort. Pathogenic HRE in the *C9ORF72* gene was present in 6.7% of cases, and disease-causing variants were detected in other known AD or FTD genes in 6.7% of cases. Furthermore, a compound heterozygous alteration of the *TREM2* gene was identified in one patient and in two patients we found RDVs in other autosomal dominantly inherited genes also associated with dementia (*CSF1R*-ALSP, *PRNP*-CJD). In two patients, the coexistence of several heterozygous RDVs in neurodegeneration-associated genes was detected (1.7%).

When we assess the diagnostic rate in this cohort we need to consider two important factors: the characteristics of the variant and the relationship of the gene with the phenotype. Based on this, we can classify patients into four groups, with different levels of certainty of a genetic diagnosis. In this sense, the diagnostic rate will depend on which group we include in the calculation as a “positive genetic test.”

Firstly, we can differentiate those cases (group 1) where a clear monogenic dementia syndrome can be diagnosed. This can be done when a pathogenic or likely pathogenic variant is detected in a known Mendelian inherited dementia gene, either heterozygous for dominant or homozygous/compound heterozygous for recessive genes. In group 2, there are patients who carry heterozygous variants in genes, in which rare variants act more as a strong genetic risk factor. In reality, the boundary between group 1 and group 2 can be blurred. Additionally, we need to analyze the relationship of the genes with the phenotype. In group 3, there are patients in whom we detect pathogenic/likely pathogenic variants in genes with the phenotype not originally associated with dementia, but still with consistent zygosity. With the widespread use of NGS large panels, this is more and more common and underlines our limited capability of differentiating patients based solely on clinical symptoms. The most questionable cases (group 4) are those patients where we identify heterozygous rare damaging variants in different genes (sometimes multiple variants in a single patient) associated with typically autosomal recessive neurodegenerative disorders.

### Group 1

Based on our experiences and on the previously published data, the detection of *C9ORF72* HRE is the first recommended step in the genetic diagnostic pipeline of the dementias [4]. This genetic alteration was the most common in our cohort since 8 patients harbored abnormal HRE in this gene. In the positive cases, the typical FTD phenotype was the most prevalent, although the clinical pictures were very diverse. Three patients reported hallucinations and one patient had developed atypical parkinsonian symptoms as well.

In familial EOAD, the alterations of *PSEN1* are most frequent [2], which was also supported by our study since we identified three RDVs in the *PSEN1* gene (p.V89L–c.265 G > C, G206S-c.616G > A, p.L166R-c.497 T > G). The c.265 G > C substitution had been previously described [17] and the pathogenic role of this alteration was supported by a functional study on the cell line of our patient (P27) [18]. At the same position, the c.265 G > T substitution resulting in the p.V89L mutation was previously reported as a damaging variant as well [26]. At position c.497 both T > C (p.L166P), c. T > G (p.L166R), (L166V), (L166del), (L166H) substitutions were reported in several EOAD patients [27–29]. In our case, the c.497 T > G (p.L166R) substitution was present. Rayn et al. identified the same substitution at this position with a similar phenotype characterized by spastic paraparesis, extrapyramidal symptoms, and behavioral changes in addition to dementia.

In the sporadic patients from our cohort, damaging rare variants were present in 16.6% of the cases. The monogenic form of dementia was identified only in 11.1% of the sporadic cases. This is in line with the results of Lacour et al. since they detected 12, 5% sporadic cases having DRVs in their cohort [30]. Sporadic cases having monogenic etiology can be explained by (1) the presence of de novo mutations, (2) the disease has autosomal recessive inheritance, and (3) the lack of information (the early loss of the parents from which the mutation was inherited or no information available about the parents) [30, 31]. Exceptionally RDVs in EOD-associated genes can act as a genetic risk factor and not the monogenic cause of the disease. For example, *PSEN2* primarily causes monogenic autosomal dominantly inherited EOAD, but one of our detected variants (p.S130L in P73) has been previously suggested to be a risk factor for neurodegeneration [19]. This variant has been already described in patients with EOAD, LOAD, or idiopathic PD [19, 32] and was lacking in our control group. Only one patient with early-onset Parkinson’s disease carried this variant in a previously studied cohort (unpublished data). Our results further support the previously published data that the *PSEN2* S130L alteration might be a potential modifying factor in neurodegenerative disorders. We assume that the coexistence of different genetic risk factors such as *PSEN2* S130L RDV, *HFE* H63F polymorphism, and *APOE* ɛ4/4 genotype may influence the AOO and the severity of the patient’s symptoms.

### Group 2

As previously described, a large proportion of EOD cases remain unexplained if only known monogenic autosomal dominant genes are examined [2]. Numerous rare variants in several genes have been identified by GWAS and NGS studies that increase the risk of AD [23]. Based on these studies, heterozygous rare coding variants have been described with moderate-to-high impact on the *TREM2*, *SORL1*, and *ABCA7* genes [23, 33]. In light of previous results, the analysis of the abovementioned risk genes in our study could increase the chance of revealing the genetic background of the EOD.

In *TREM2*, compound heterozygous alterations were detected in one patient associated with NHD. The NHD was first described with progressive dementia and pathological bone fractures [34]. In the last few years, several cases were reported with EOD and without bone cysts [21, 34]. In most cases, the FTD phenotype was associated, but in some cases the AD-like phenotype and/or morphological alterations were reported in association with *TREM2* gene mutations [34, 35]. Dementia coexisted in most of the cases with epilepsy, like in our patient. The heterozygous p.R47H variant was previously described as a genetic risk factor for AD [34]. It was identified in two unrelated cases (1.7%) in our cohort. In addition to heterozygous *TREM*2 RDVs, the rare variants of other genes have been described as a moderate-to-high-risk factor for AD: *ABCA7* and *SORL1* [23, 33]. The heterozygous RDVs in these genes were detected in four patients that were absent in our control group. P35 carried two additional possible RDVs in the *LRRK2* (p.A1862V) and *SPG11* (p.V2053M) genes.

### Group 3

The presence of the heterozygous pathogenic variant in the *CSF1R* gene indicated the diagnosis of ALSP. The neuropathology of ALSP caused by the *CSF1R* gene alteration largely overlaps with a dementia-related disease, e.g., AD and FTD [3–5] which could lead to misdiagnosis. However, several studies suggest that in FTD spectrum disease not only clinical symptoms but also genetic background may overlap with ALSP [3, 36]. Our results also support this observation: in our case, the clinical phenotype corresponded to the FTD, and we could find the signs of ALSP on the MRI of the referred patients after the genetic testing of the reverse phenotype. Furthermore, due to slow progression and non-prion typical symptoms, the young P112 was enrolled in our cohort. A RVD *PRNP* was found which could cause a rare but slow-acting variant of prion disease that is specifically characteristic for certain genetic mutations [22]. These cases strongly emphasize the importance of developing a more comprehensive multi-gene NGS panel for neurodegenerative diseases and the introduction of this to the routine diagnostic workflow.

### Group 4

Several RDVs were identified in genes previously associated with other neurodegenerative disorders. Because of the similar pathomechanism of AD and PD [5], variants of genes known to cause monogenic PD (*LRRK2*, *PINK1*, *PRNK*) might contribute to the AD patient’s symptoms. Furthermore, several studies reported variants in PD-associated genes in AD patients [5, 37]. In our NGS study, we identified RDVs in genes which were previously associated with other neurodegenerative disorders with or without dementia, such as *C19ORF12*, *PRKN*, *LRRK2*, and *PARK7* genes in three patients (P81, P35, P50). In two of these cases, the detected RDVs coexisted (P50: *C19ORF12* and *PARK7* RDVs, P35: *ABCA7*; *LRRK2* and *SPG11* RDVs). Both of them showed prefrontal symptoms with parkinsonism. An increasing number of studies support that the heterozygous variants of the *C19ORF12* gene might play a role in disease development either by increasing the risk or causing monogenic disorders [38, 39]. Furthermore, monoallelic alterations could be associated with PD-like symptoms as well [39]. As *PARK7* is suggested to cause dementia with Lewy bodies (DLB) [5, 40], the coexistence of variants in *C19ORF12* and *PARK7* is assumed to contribute to the symptoms of P50. In P35 the coexistence of RDVs of an AD susceptibility gene (*ABCA7*), a PD-related gene (*LRRK2*) and an ALS-related gene (*SPG11*) were detected. Based on recent studies which suggested that both the *LRRK2* and *SPG11* genes are associated with an AD/FTD-like phenotype [5, 41], the development of the complex phenotype was assumed to be due to the co-occurrence of the three RDVs. Considering that these genes showed various degrees of correlation with dementia, in addition to the examination of both their protein functions, epidemiological studies with larger sample sizes could further clarify their potential role in the pathomechanism of degenerative dementia.

P52 carried a heterozygous likely pathogenic variant in the *PSAP* gene which was previously associated with leukodystrophy in the autosomal recessive form [42]. Previous studies found that in patients with *GRN* mutations the PSAP level was decreased in neurons. In transgenic mice with reduced PSAP expression, FTD-like pathology and behavioral changes were observed which were similar to those in mice with *GRN* mutations [43]. Since *PSAP* and *GRN* are both lysosomal genes, we hypothesize that biallelic rare variants of these genes are causing severe rare diseases, while the monoallelic rare variants may play role in the development of common neurodegenerative diseases. Homozygous or compound heterozygous mutations of the *GRN* gene cause *CLN11* (ceroid lipofuscinosis neuronal), while the heterozygous variant of the same gene is associated with FTD. A similar situation is observed in association with the *GBA*. Biallelic RDVs of the *GBA* are associated with Gaucher disease, while monoallelic forms are strongly associated with PD [8]. The *PSAP* gene, which causes SAP deficiency disease in homozygous form, could aggravate neurodegenerative processes in the heterozygous form [43]. In light of this data, we hypothesized that the p.E108V substitution in the P52 case might contribute to the risk of dementia. This observation further emphasizes the relationship between genes involved in lysosomal function and neurodegeneration.

In various neurodegenerative diseases, several clinical and pathological similarities could be observed. Based on these overlaps, it is hypothesized that their genetic background may also have common features. This explains why the clinical picture may sometimes lead to misdiagnosis. Furthermore, various clinical phenotypes could be observed in different types of dementia, but there are also overlaps between them and other neurodegenerative disorders; thus, it can be a challenge to diagnose them based on the phenotype alone [1]. Therefore, comprehensive genetic testing which can simultaneously examine all genes associated with neurodegeneration could greatly accelerate the path to achieve the proper genetic diagnosis, especially in early-onset forms. It can also reveal hidden correlations and overlaps in neurodegenerative diseases that would remain concealed in separate genetic testing.

## Supplementary Information

Below is the link to the electronic supplementary material.

Supplementary file1 (DOCX 26.0 KB)

## Data Availability

The raw data supporting the conclusions of this manuscript will be made available by the authors, without undue reservation, to any qualified researcher.
