# Supplementary material for: Genetic landscape of early-onset dementia in Hungary
Source: Neurol Sci. 2022 Jun 25;43(9):5289–300. doi: 10.1007/s10072-022-06168-8 (PMC9385840; doi:10.1007/s10072-022-06168-8)
Supplement: Supplementary file 1 — Supplementary file1 (DOCX 26.0 KB) [file 10072_2022_6168_MOESM1_ESM.docx]

**Genetic landscape of early onset dementia in Hungary**

**Neurological Sciences**

Dora Csaban^1^, Anett Illes^2^, Toth-Bencsik Renata^1^, Peter Balicza^1^, Klara Pentelenyi^1^, Viktor Molnar^1^, Andras Gezsi^3^, Zoltan Grosz^1^, Aniko Gal^1^, Tibor Kovacs^4^, Peter Klivenyi^5^, Maria Judit Molnar^1^*

^1^Institute of Genomic Medicine and Rare Disorders, Semmelweis University, H- 1082 Budapest, Hungary

^2^PentaCore Laboratory Budapest, Budapest, Hungary

^3^Department of Measurement and Information Systems, Budapest University of Technology and Economics, Budapest, Hungary

^4^Department of Neurology, Semmelweis University, Budapest, Hungary

^5^Department of Neurology, Faculty of Medicine, Albert Szent-Györgyi Clinical Center, University of Szeged, Szeged, Hungary

*Correspondence: Maria Judit Molnar [molnar.mariajudit@med.semmelweis-univ.hu](mailto:molnar.mariajudit@med.semmelweis-univ.hu)
ORCID ID: 0000-0001-9350-1864

**Supplementary Table 1. List of genes which were investigated**

| **Gene symbol** | **Gene name** | **Method** |
| --- | --- | --- |
| ABCA7 | ATP binding cassette subfamily A member 7 | NGS |
| ABI3 | ABI Family Member 3 | NGS |
| ADAM10 | ADAM metallopeptidase domain 10 | NGS |
| ADORA1 | adenosine A1 receptor | NGS |
| AKT1 | AKT serine/threonine kinase 1 | NGS |
| ALS2 | alsin Rho guanine nucleotide exchange factor ALS2 | NGS |
| ANG | angiogenin | NGS |
| APOE | apolipoprotein E | NGS; RFLP |
| APP | amyloid beta precursor protein | NGS; Sanger sequencing |
| APTX | Aprataxin | NGS |
| ATM | ATM serine/threonine kinase | NGS |
| ATP13A2 | ATPase cation transporting 13A2 | NGS |
| ATP1A3 | ATPase Na+/K+ transporting subunit alpha 3 | NGS |
| ATP6AP2 | ATPase H+ transporting accessory protein 2 | NGS |
| ATXN2 | ataxin 2 | NGS |
| BACE1 | beta-secretase 1 | NGS |
| BIN1 | bridging integrator 1 | NGS |
| C19ORF12 | chromosome 19 open reading frame 12 | NGS |
| C21ORF2 | chromosome 21 open reading frame 2 | NGS |
| C9ORF72 | chromosome 9 open reading frame 72 | RP-PCR; NGS |
| CASS4 | Cas Scaffold Protein Family Member 4 | NGS |
| CD2AP | CD2 associated protein | NGS |
| CD33 | CD33 molecule | NGS |
| CELF1 | CUGBP Elav-Like Family Member 1 | NGS |
| CFL1 | cofilin 1 | NGS |
| CHCHD10 | coiled-coil-helix-coiled-coil-helix domain containing 10 | NGS |
| CHCHD2 | coiled-coil-helix-coiled-coil-helix domain containing 2 | NGS |
| CHMP2B | charged multivesicular body protein 2B | NGS |
| CLU | clusterin | NGS |
| COASY | Coenzyme A Synthase | NGS |
| CP | ceruloplasmin | NGS |
| CR1 | complement C3b/C4b receptor 1 | NGS |
| CTSD | cathepsin D | NGS |
| CSF1R | colony stimulating factor 1 receptor | NGS |
| CST3 | cystatin C | NGS |
| DCAF17 | DDB1 and CUL4 associated factor 17 | NGS |
| DCC | DCC netrin 1 receptor | NGS |
| DCTN1 | dynactin subunit 1 | NGS |
| DNAJC13 | DnaJ heat shock protein family (Hsp40) member C13 | NGS |
| DNAJC5 | DnaJ heat shock protein family (Hsp40) member C5 | NGS |
| DNAJC6 | DnaJ heat shock protein family (Hsp40) member C6 | NGS |
| DNAL4 | Dynein Axonemal Light Chain 4 | NGS |
| DNMT1 | DNA methyltransferase 1 | NGS |
| EIF4G1 | eukaryotic translation initiation factor 4 gamma 1 | NGS |
| ELAVL1 | ELAV like RNA binding protein 1 | NGS |
| EPHA1 | EPH receptor A1 | NGS |
| EPHA4 | EPH receptor A4 | NGS |
| ERBB4 | erb-b2 receptor tyrosine kinase 4 | NGS |
| FA2H | Fatty Acid 2-Hydroxylase | NGS |
| FBXO7 | F-box protein 7 | NGS |
| FIG4 | FIG4 phosphoinositide 5-phosphatase | NGS |
| FTL | ferritin light chain | NGS |
| FUS | FUS RNA binding protein | NGS |
| GBA | glucosylceramidase beta | NGS |
| GCH1 | GTP cyclohydrolase 1 | NGS |
| GIGYF2 | GRB10 interacting GYF protein 2 | NGS |
| GRN | granulin precursor | NGS; Sanger sequencing |
| HNRNPA1 | heterogeneous nuclear ribonucleoprotein A1 | NGS |
| HNRNPA2B1 | Heterogeneous Nuclear Ribonucleoprotein A2/B1 | NGS |
| HTRA2 | HtrA serine peptidase 2 | NGS |
| ITM2B | Integral Membrane Protein 2B | NGS |
| LRRK2 | leucine rich repeat kinase 2 | NGS |
| MAPT | microtubule associated protein tau | NGS; Sanger sequencing |
| MARK2 | microtubule affinity regulating kinase 2 | NGS |
| MARK4 | Microtubule Affinity Regulating Kinase 4 | NGS |
| MATR3 | Matrin 3 | NGS |
| MS4A4A | Membrane Spanning 4-Domains A4A | NGS |
| MS4A4E | membrane spanning 4-domains A4E | NGS |
| MS4A6A | membrane spanning 4-domains A6A | NGS |
| MS4A6E | Membrane Spanning 4-Domains A6E | NGS |
| NEFH | Neurofilament Heavy Chain | NGS |
| NEU1 | Neuraminidase 1 | NGS |
| NOTCH3 | notch receptor 3 | NGS |
| OPTN | optineurin | NGS |
| PANK2 | pantothenate kinase 2 | NGS |
| PARK2 | parkin RBR E3 ubiquitin protein ligase | NGS |
| PARK7 | Parkinsonism associated deglycase | NGS |
| PFN1 | Profilin 1 | NGS |
| PICALM | phosphatidylinositol binding clathrin assembly protein | NGS |
| PINK1 | PTEN induced kinase 1 | NGS |
| PLA2G6 | phospholipase A2 group VI | NGS |
| POLG | DNA polymerase gamma, catalytic subunit | NGS |
| PRKRA | protein activator of interferon induced protein kinase | NGS |
| PRNP | prion protein | NGS; Sanger sequencing |
| PRPH | Peripherin | NGS |
| PSAP | prosaposin | NGS |
| PSEN1 | presenilin 1 | NGS; Sanger sequencing |
| PSEN2 | presenilin 2 | NGS; Sanger sequencing |
| PTK2B | protein tyrosine kinase 2 beta | NGS |
| RAD51 | RAD51 recombinase | NGS |
| SCP2 | Sterol Carrier Protein 2 | NGS |
| SETX | Senataxin | NGS |
| SIGMAR1 | Sigma Non-Opioid Intracellular Receptor 1 | NGS |
| SLC30A10 | solute carrier family 30 member 10 | NGS |
| SLC6A3 | solute carrier family 6 member 3 | NGS |
| SNCA | synuclein alpha | NGS |
| SNCB | synuclein beta | NGS |
| SOD1 | superoxide dismutase 1 | NGS |
| SORL1 | sortilin related receptor 1 | NGS |
| SPG11 | SPG11 vesicle trafficking associated, spatacsin | NGS |
| SPR | sepiapterin reductase | NGS |
| SQSTM1 | sequestosome 1 | NGS |
| SYNJ1 | synaptojanin 1 | NGS |
| TAF1 | TATA-box binding protein associated factor 1 | NGS |
| TARDBP | TAR DNA binding protein | NGS |
| TBK1 | TANK binding kinase 1 | NGS |
| TBP | TATA-box binding protein | NGS |
| TFG | Trafficking From ER To Golgi Regulator | NGS |
| TH | tyrosine hydroxylase | NGS |
| THAP1 | THAP Domain Containing 1 | NGS |
| TMEM106B | transmembrane protein 106B | NGS |
| TMEM230 | transmembrane protein 230 | NGS |
| TOMM40 | translocase of outer mitochondrial membrane 40 | NGS |
| TOR1A | torsin family 1 member A | NGS |
| TPP1 | tripeptidyl peptidase 1 | NGS |
| TREM2 | triggering receptor expressed on myeloid cells 2 | NGS |
| TUBA4A | Tubulin Alpha 4a | NGS |
| TYROBP | TYRO protein tyrosine kinase binding protein | NGS |
| UBE3A | ubiquitin protein ligase E3A | NGS |
| UBQLN2 | Ubiquilin 2 | NGS |
| UCHL1 | ubiquitin C-terminal hydrolase L1 | NGS |
| UNC13A | Unc-13 Homolog A | NGS |
| VAPB | VAMP associated protein B and C | NGS |
| VCP | valosin containing protein | NGS |
| VPS13C | vacuolar protein sorting 13 homolog C | NGS |
| VPS35 | VPS35 retromer complex component | NGS |
| WDR45 | WD repeat domain 45 | NGS |

NGS next-generation sequencing

RFLP restriction fragment length polymorphism

RP-PCR repeat-primed PCR
